# Data of the quantification of the influence of DMSO concentration on the kinetic of interaction between TNFα and SPD304

**DOI:** 10.1016/j.dib.2021.107610

**Published:** 2021-11-20

**Authors:** Ghada Attia, Aïda Mascret, Chouki Zerrouki, Najla Fourati, Maité Sylla-Iyarreta Veitía, Marc Port

**Affiliations:** a Laboratoire SATIE, UMR CNRS 8029, Conservatoire National des Arts et Métiers (Cnam), 292 rue Saint Martin, HESAM Université, Paris 75003, France; b Equipe de Chimie Moléculaire du Laboratoire Génomique, Bioinformatique et Chimie Moléculaire (EA 7528), Conservatoire National des Arts et Métiers (Cnam), 2 rue Conté, HESAM Université, Paris 75003, France; c Peptinov, Pépinière Cochin Santé, Hôpital Cochin, 29 rue du Faubourg Saint Jacques, Paris 75014, France

**Keywords:** SPD304, Surface acoustic wave biosensor, TNFα, Affinity, DMSO cosolvent

## Abstract

The data presented here are related to the article entitled “New contributions to the drug profile of TNFα inhibitor SPD304: affinity, selectivity ADMET considerations” published in the European Journal of pharmacology. As DMSO is usually used as a co-solvent to dissolve low aqueous soluble small molecules, such as SPD304, we have investigated the influence of its concentration on the kinetic of interaction between tumor necrosis factor α (TNF-α) and its inhibitor, SPD304. The presented data, acquired using a surface acoustic wave sensor, can be used in further biological studies to compare the kinetic of interaction between proteins/small molecules in general and TNFα/inhibitors in particular. The estimated dissociation constant can be compared to other ones to statute on the degree of affinity between a protein and a given molecule.

## Specifications Table

**Table:** 

Subject	Physical and Theoretical Chemistry
Specific subject area	Effects of DMSO cosolvent on TNFα/SPD304 interaction
Type of data	Figures
How data were acquired	The data were acquired using a surface acoustic wave sensor which consists in dual delay lines fabricated on 36°rot lithium tantalate piezoelectric substrate. The interdigital transducers, realized by evaporation of (20/80) nm Cr/Au layers, were photolithographically patterned with a periodicity of λ = 40 µm which corresponds to an operating frequency of about 103 MHz. The measurement setup consists of a TNFa grafted SAW sensor, a Kalrez® flow cell, a PMMA cover including inletsand outlets connected to a Gilson® 3 peristaltic pump, and a HP8214 network analyser.
Data format	Raw and analysed graphs. The presented results are average of triplicate sample analysis.
Parameters for data collection	A HP8214 Network analyser was used to monitor phase output signals versus time at a fixed frequency.
Description of data collection	Temporal phase and amplitude output signals’ variations, after functionalization steps and further analytes’ injections, were monitored by a HP8214 network analyzer piloted by a home-made software
Data source location	*Conservatoire National des Arts et Métiers (Cnam), Paris, France.*
Data accessibility	Data are available with the article
Related research article	for co-submission manuscripts Aïda Mascret, Hadley Mouhsine, Ghada Attia, Damien Cabrera, Mohamed Benchekroun, Patrick Gizzi, Chouki Zerrouki, Najla Fourati, Jean-François Zagury, Maité Sylla-Iyarreta Veitia Sylla, Marc Port. New contributions to the drug profile of TNFa inhibitor SPD 304: affinity, selectivity and ADMET considerations. European Journal of pharmacology, 907, 174285, 2021, https://doi.org/10.1016/j.ejphar.2021.174285

## Value of the Data

• Dimethyl sulfoxide (DMSO) is a cosolvent often used for biological or biophysical characterization. In this context, it is important to know whether its presence can affect the interaction between small molecules inhibiting TNFα and the latter.
• Research is currently underway to find small molecules that inhibit TNFα to overcome the limits of biomolecules currently on the market. To develop this research, it is essential to be able to accurately measure the affinity of these small molecules towards TNFα. Alongside conventional devices, surface acoustic wave (SAW) sensors prove to be an accurate and powerful tool to support such investigations.
• Due to their high sensitivity and versatility, SAW sensors can be consider for reliable and rapid screening of many molecules before considering only the promising ones for biological tests.
• The (6,7-dimethyl-3-[[methyl[2-[methyl[[1-[3-(trifluoromethyl)phenyl]-1H-indol-3-yl]methyl]amino]ethyl]amino]methyl]-(4H-1-benzopyran-4-one dihydrochloride) in a salt form (SPD304) molecule is considered as a reference molecule in almost all publications describing direct inhibitors of TNFα. He *et al* in 2005, using a fluorescence homoquenching-based assay, postulated that the mechanism was described by a predissociation-independent model where SPD304 interacts with TNFα trimer to form an intermediate complex which promotes TNFα monomer dissociation.

## 1 Data Description

**Fig. 1:**
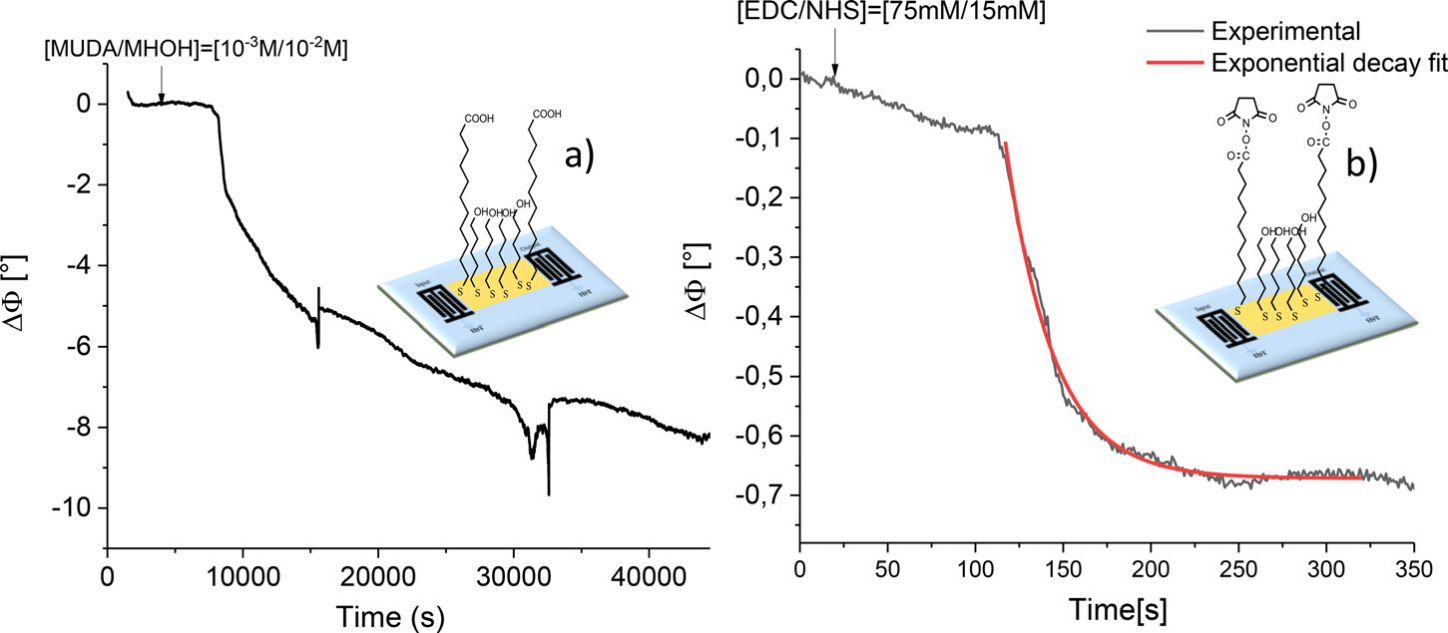
Schematic representation and phase variations versus time after (a) SAMs grafting, (b) activation of the carboxylic acid groups with EDC/NHS.

**Fig. 2:**
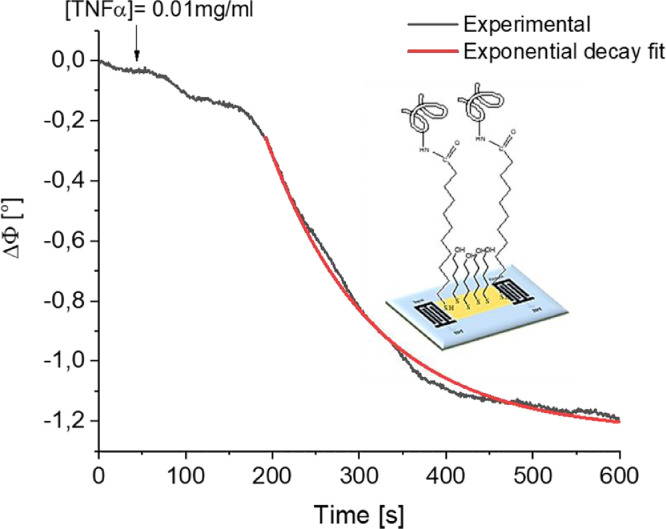
Schematic representation and phase variations versus time after the injection of TNFα protein.

The following figures are related to the design of the SAW biosensor and SPD304 detection in absence and in presence of dimethyl sulfoxide (DMSO). Representations of temporal phase variations after the grafting of the 11-mercaptoundecanoic acid (MUDA) / 6-mercapto-1-hexanol (MHOH) (1/9, v/v) self-assembled monolayers (SAMs) and further activation of the carboxylic acid groups with 1-ethyl-3-[3-dimethylaminopropyl] carbodiimide hydrochloride (EDC) / N-hydroxysuccinimide (NHS) are presented in Fig. 1a and 1b, respectively. This functionalisation step is followed by TNFα protein anchoring. The corresponding phase variation versus time graph is depicted in Fig. 2.

**Fig. 3:**
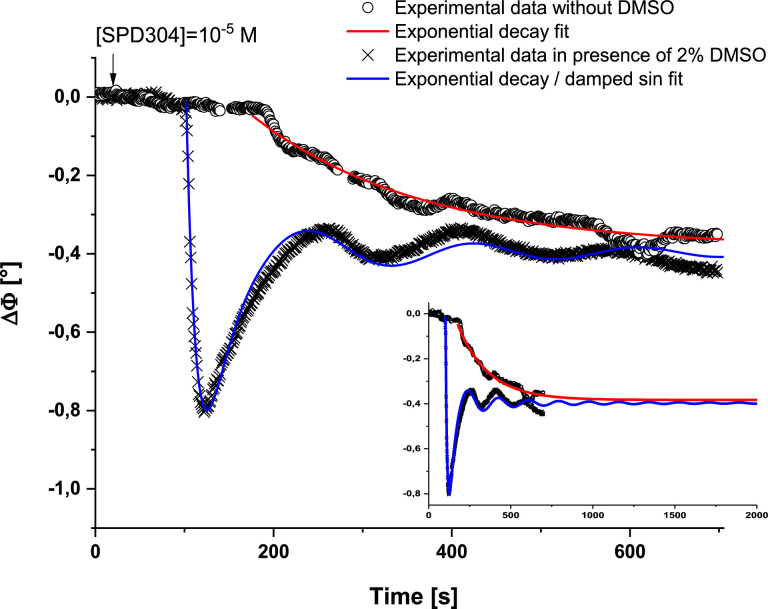
Phase shift variation versus time after the injection of 10^−5^ M of SPD304 in absence and in presence of 2% of DMSO and their further modelling with exponential decay function (in the absence of DMSO) and a combined decay / exponential decay-damped sine functions (in presence of 2% DMSO). Inset: extrapolation of the two models over a sufficiently long period for the disappearance/reduction of the DMSO effect.

**Fig. 4:**
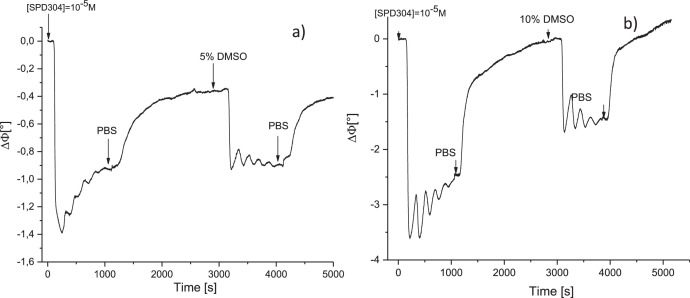
Phase variations versus time after the injections of SPD304 dissolved in PBS solutions containing a) 5% of DMSO, b) 10% of DMSO.

Figs. 3 and 4 are related to the effect of DMSO cosolvent concentration on the kinetic of interaction between TNFα and SPD304. In this study, we have chosen to investigate three concentrations of DMSO: 2%, 5% and 10% after the injection of the same concentration of SPD304 (10^−5^ M) in the flow cell.

## 2 Primary Data

All the raw data corresponding to these figures are gathered in the Supplementary Information.

Table S1a and b contain the primary data related to the functionalization of the SAW sensing area with MUDA/MHOH and further activation with EDC/NHS.

Primary data concerning TNFα grafting are gathered in Table S2. Those related to the detection of SPD304 in the absence and in the presence of 2% DMSO are collected in the Tables S3a and 3b, respectively.

The primary data collected after the injection of SPD304 in DMSO at a concentration of 5% and 10% are collected in Table S4.

## 3 Experimental Design, Materials and Methods

### 3.1 Validation of surface functionalization of biosensor

The surface functionalization step was validated carefully to ensure reproducibility of the biosensor. Phase variations versus time after mixed SAMs grafting and their further activation with EDC/NHS are gathered in Fig. 1. Fig. 1a show that the phase output signal decreases slowly after SAMs injection and that it stabilizes after 40,000 s. Almost 11 h are thus needed for the organization and the formation of the mixed self-assembled monolayers of (MUDA/MHOH) on SAW gold surface. This duration is in phase with those reported in literature [1,2].

The device was after that rinsed with ethanol, to remove un-grafted molecules, and then with PBS. When phase stability was reached, a continuous flow of EDC/NHS was injected on the SAW sensing area. Results presented in Fig. 1b show that the formation of NHS ester was completed in approximatively 5 min. A further fitting of phase variations versus time with an exponential decay function indicates that the characteristic time was of order of (27 ± 1) s confirming that the kinetics of activation reaction is fast. Besides the large difference in terms of reaction duration, Fig. 1a and b also reveal that the phase shift variations are much higher for SAMs grafting than for EDC/NHS activation. This can be explained by the fact that the self-assembled layers cover the entire sensitive area and they are directly linked to it.

TNFα protein was after that grafted on the SAMs via a carbodiimide coupling reaction. The corresponding temporal phase variations curve (presented in Fig. 2.) indicate that the phase signal was stabilized approximatively 600 s after TNFα injection. The characteristic time constant τc, estimated with a modelling with an exponential decay function, was of order of (123 ± 1) s.

### 3.2 The effect of DMSO cosolvent concentration

The effect of DMSO cosolvent concentration on the kinetic of interaction between TNFα and SPD304 was then investigated. Experiments were performed in the absence and in presence of 2%, 5% and 10% of DMSO. For all these experiments, we have chosen to inject the same concentration of SPD304 (10^−5^ M) in the flow cell, so that only the effect of DMSO is highlighted. Temporal phase shifts variations after the injection of the ligand in the absence and in the presence of 2% of DMSO are plotted in Fig. 3. Results show that the injection of SPD304 dissolved in a PBS solution engenders only a phase decrease, while, we have recorded, in presence of 2% of DMSO, a large phase decrease, followed first by phase increase and then by damped oscillations, prior to phase output signal stabilization at almost the same value as that of SPD304 in PBS. The shapes of the two graphs are clearly different, but the phase shifts following the injection of the same concentration of SPD304 are quasi-comparable, once the stability reached.

For better comprehension of DMSO effect, we have fitted both gravimetric curves with exponential decay functions (first order for PBS solution, and first order to which we added a damped sine for 2% DMSO solution) to extract their corresponding time constants τ. Results indicate that τ _SPD304 in 2% DMSO_ = (17.5 ± 0.5) s is largely inferior to that of τ _SPD304 inPBS_ = (176 ± 7) s. Adding 2% of DMSO cosolvent permits thus to accelerate the kinetic of interaction between TNFα and SPD304. Extrapolation of the two models over a sufficiently long period, until the disappearance/reduction of the effect of DMSO, shows the same frequency shift within uncertainties (the relative difference is lower than 4%).

Phase variations versus time after the injection of SPD304 dissolved in PBS solutions, containing 5% and 10% of DMSO are presented in Fig. 4.

Fig. 4a shows that the phase decreases drastically to 1.4°, and then increases slowly with damped oscillations till ≈ 1°. A further rinsing with PBS permits to eliminate the excess of cosolvent molecules, and the phase reaches after that the same value as that of SPD304 in PBS. Here also, we can conclude that adding 5% of DMSO cosolvent permits only to accelerate the kinetic of interaction between TNFα and SPD304. On the other hand, this was not the case for SPD304 dissolved in 10% of DMSO, as the phase signal returns to its initial value after rinsing with PBS. At this high value of DMSO concentration, we have probably destabilized and dissociated TNFα protein, confirming thus the results of Corti et al [3].

DMSO, added at moderate concentrations (< 5%), permitted to accelerate the kinetic of binding between the protein and the ligand. However, at higher concentrations (10%) the protein is denatured leading to the inhibition of TNFα/SPD304 interaction.

## CRediT authorship contribution statement

**Ghada Attia:** Methodology, Investigation, Data curation, Formal analysis. **Aïda Mascret:** Methodology, Investigation, Resources. **Chouki Zerrouki:** Validation, Data curation, Supervision. **Najla Fourati:** Conceptualization, Validation, Supervision, Writing – original draft. **Maité Sylla-Iyarreta Veitía:** Writing – review & editing, Visualization, Supervision. **Marc Port:** Supervision, Project administration, Funding acquisition.

## Declaration of Competing Interest

The authors declare that they have no known competing financial interests or personal relationships which have, or could be perceived to have, influenced the work reported in this article.

## Appendix Supplementary Materials

Image, application 1
